# Impact of a novel homozygous mutation in nicotinamide nucleotide transhydrogenase on mitochondrial DNA integrity in a case of familial glucocorticoid deficiency

**DOI:** 10.1016/j.bbacli.2014.12.003

**Published:** 2014-12-13

**Authors:** Yasuko Fujisawa, Eleonora Napoli, Sarah Wong, Gyu Song, Rie Yamaguchi, Toshiharu Matsui, Keisuke Nagasaki, Tsutomu Ogata, Cecilia Giulivi

**Affiliations:** a Department of Pediatrics, Hamamatsu University School of Medicine, Hamamatsu 431-3192, Japan; b Department of Molecular Biosciences, University of California Davis, Davis, CA 95616, USA; c Department of Pediatrics, Nagaoka Chuo General Hospital, Nagaoka 940-8653, Japan; d Division of Pediatrics, Niigata University Graduate School of Medicine and Dental Sciences, Niigata 951-8122, Japan; e Medical Investigations of Neurodevelopmental Disorders (M. I. N. D.) Institute, University of California Davis, Sacramento, CA 95616, USA

**Keywords:** Familial glucocorticoid deficiency, Mitochondrial biogenesis, Mitochondrial replication, Nicotinamide nucleotide transhydrogenase, Oxidative phosphorylation, Oxidative stress

## Abstract

**Background:**

Familial glucocorticoid deficiency (FGD) is a rare autosomal recessive disorder that is characterized by isolated glucocorticoid deficiency. Recently, mutations in the gene encoding for the mitochondrial nicotinamide nucleotide transhydrogenase (NNT) have been identified as a causative gene for FGD; however, no NNT activities have been reported in FGD patients carrying NNT mutations.

**Methods:**

Clinical, biochemical and molecular analyses of lymphocytes from FDG homozygous and heterozygous carriers for the F215S NNT mutation were performed.

**Results:**

In this study, we described an FGD-affected Japanese patient carrying a novel NNT homozygous mutation (c.644T>C; F215S) with a significant loss-of-function (NNT activity = 31% of healthy controls) in peripheral blood cells' mitochondria. The NNT activities of the parents, heterozygous for the mutation, were 61% of the controls.

**Conclusions:**

Our results indicated that (i) mitochondrial biogenesis (citrate synthase activity) and/or mtDNA replication (mtDNA copy number) were affected at ≤ 60% NNT activity because these parameters were affected in individuals carrying either one or both mutated alleles; and (ii) other outcomes (mtDNA deletions, protein tyrosine nitration, OXPHOS capacity) were affected at ≤ 30% NNT activity as also observed in murine cerebellar mitochondria from C57BL/6J (*NNT*^−*/*−^) vs. C57BL/6JN (*NNT*^+*/*+^) substrains.

**General significance:**

By studying a family affected with a novel point mutation in the NNT gene, a gene–dose response was found for various mitochondrial outcomes providing for novel insights into the role of NNT in the maintenance of mtDNA integrity beyond that described for preventing oxidative stress.

## Highlights

• Mutations in *NNT* were reported for a subset of FGD patients.
• A gene–dose effect is reported for a novel *NNT* pathogenic mutation (c.644T > C).
• Homozygous carrier (FGD patient) has negligible NNT activity.
• Patient exhibited OXPHOS deficits with oxidative damage to mtDNA and proteins.
• Heterozygous carriers (parents) were asymptomatic with ~ 60% NNT activity.
• All carriers showed deficits in mitochondrial biogenesis and mtDNA replication.

## 1 Introduction

Familial glucocorticoid deficiency (FGD) is a rare autosomal recessive disorder that is characterized by isolated glucocorticoid deficiency [1] reported for the first time in 1959 [2]. The diagnosis of FGD is based on clinical findings with patients usually presenting hypoglycemia, seizures, jaundice, hyperpigmentation, failure to thrive, muscle weakness and frequent or severe infections. The biochemical findings are a markedly elevated plasma ACTH in the presence of low cortisol but with a preserved mineralocorticoid production and a characteristic ACTH insensitivity [3], [4], [5], [6], [7], [8], [9], [10], [11], [12], [13], [14], [15]. The long-term neurological consequences of FGD can vary from learning difficulties, mild dementia [6], spastic quadriplegia [16], [17], which may reflect the severity and number of hypoglycemic episodes during childhood [17]. Although this disease is easily treatable when recognized, hypoglycemic attacks and convulsions may result in coma or even death [18].

FGD has been identified with mutations in the melanocortin-2 receptor gene (*MC2R*) (~ 25% of patients), the *MRAP* gene encoding the MC2R accessory protein (~ 20% of patients), the steroidogenic acute regulatory gene (*STAR*; ~ 5% of patients), and by a single mutation in the mini-chromosome maintenance-deficient 4 homolog gene (*MCM4*) in the Irish Traveler population [reviewed in ref [15]]. Although half of the FGD cases are caused by mutations in genes involved in (i) the ACTH signaling–steroidogenic pathway (*MC2R*, *MRAP*, *STAR*), (ii) the replicative complex critical for normal DNA replication and genome stability (*MCM4*), and (iii) antioxidant system (*TXNRD2*) [19], no mutations have been found in the rest of the cases, suggesting the presence of hitherto unidentified responsible genes in FGD. Recently, mutations in the *NNT* gene, encoding the mitochondrial nicotinamide nucleotide transhydrogenase (NNT), have been identified as a causative gene for FGD [20]. Although a clear link between glucocorticoid deficiency and NNT deficits has not been presented yet, it can be hypothesized that the reducing equivalents required for the synthesis of glucocorticoids via the adrenodoxin oxidoreductase system could be supplied by NNT given the mitochondrial localization. The adrenodoxin oxidoreductase comprises a short electron transport chain that provides reducing equivalents for biosynthesis of iron–sulfur clusters ([21]) and steroid hormones [22], [23], [24], serving as the first electron transfer protein in all the mitochondrial P450 systems, including cholesterol side chain cleavage in all steroidogenic tissues, steroid 11-beta hydroxylation in the adrenal cortex, 25-OH-vitamin D3-24 hydroxylation in the kidney, and sterol C-27 hydroxylation in the liver (Eq. (1)):

(1) $$2\;\mathrm{oxidized}\;\mathrm{adrenodoxin}\;+\;\mathrm{NADPH}\;\to \;2\;\mathrm{reduced}\;\mathrm{adrenodoxin}\;+\;{\mathrm{NADP}}^{+}\text{.}$$

NNT is located at the inner mitochondrial membrane of eukaryotes and in bacteria. Under physiological conditions, this transporter generates reduced nicotinamide adenine dinucleotide phosphate (NADPH) and NAD^+^ from NADP^+^ and NADH in the mitochondrial matrix fueled by the proton gradient [25], [26], [27] (Eq. (2)).

(2) $$H_{\mathrm{out}}^{+}\;+\;\mathrm{NADH}\;+\;{\mathrm{NADP}}^{+}\;\to H_{\mathrm{in}}^{+}\;+\;{NAD}^{+}+\;\mathrm{NADPH}\text{.}$$

The direction of proton movement is the same as that for the F_1_F_O_-ATP synthase, where Δp favors the forward reaction (left to right; the *Keq* for the non-energy linked NNT activity is 0.79 and that of the energy-linked is 480 [28], [29]). The apparent equilibrium shifts towards NADPH formation through a conformationally-driven mechanism [26]. NNT activity in rat brain has been estimated to account for ~ 40% of the total cellular supply of NADPH, with the remainder provided by the pentose phosphate pathway, malic enzyme and NADP-dependent isocitrate dehydrogenase [30].

NADPH is a co-substrate for glutathione reductase (GR) and thioredoxin reductase [30], [31], [32], [33]. These enzymes are part of an important antioxidant defense mechanism that catalyzes the reduction of lipid peroxides or hydrogen peroxide to the corresponding alcohol or water, preventing the formation of other, more reactive oxygen species (ROS) such as hydroxyl or hydroperoxyl radicals [31]. Loss of NNT activity in a variety of biological models is associated with a lower supply of mitochondrial NADPH, increased mitochondrial ROS production and decreased [GSH]/[GSSG] ratio [34], making mitochondria more susceptible to ROS-induced damage [20], [34], [35]. A substrain of C57BL/6J mice, carrying a spontaneous missense (M to T) mutation in the mitochondrial leader sequence of NNT coupled with an in-frame five exon deletion removing four putative transmembrane helices [36], results in an almost negligible NNT activity [37] exhibiting a phenotype consistent with FGD [20]. In addition, these mice show an impaired glucose homeostasis and insulin release from β-cells by high glucose compared to other mouse strains [36], [38] and altered susceptibility to diet-induced obesity [39]. Adrenal glands from these mice have increased cellular apoptosis in zona fasciculata and compromised basal and stimulated corticosterone levels [20].

Taken together, these reports suggest that losses in NNT activity could result in (i) a defective response to cope with oxidative stress, (ii) deficits in mitochondrial NADPH production which, depending on the tissue, will lead to a defective steroidogenesis, and (iii) a combination of both effects. However, there are two significant aspects of this field that have not been studied in detail. One, despite the 21 different *NNT* mutations reported in 15 kindred with FGD predicted to significantly alter either the targeting of NNT to mitochondria or its activity [15], no direct functional studies of NNT activity have been performed in FGD patients carrying *NNT* mutations. Thus, a clear correlation between genotype–phenotype has not been explored. The other issue is that increases in biomarkers of oxidative stress observed in some NNT-deficient biological models may have obscured the role of NNT as an NADPH provider for relevant pathways other than those coping with oxidative stress such as mitochondrial DNA (mtDNA) replication.

In this regard, mtDNA replication and maintenance require the presence of a suitable dNTP pool, especially dTTP [40], [41], [42], whose synthesis depends on 3 enzymes, serine hydroxymethyltransferase-2, thymidylate synthase and dihydrofolate reductase (DHFR), in which the last one is NADPH- and folate-dependent. Rats fed a folate-deficient diet for 2 weeks showed significant increases in the mtDNA^4834^ deletion in lymphocytes [43]. By 4 weeks, increases in mtDNA copy number in lymphocytes and skeletal muscle [43], increased hepatic oxidative stress, lower CCO activity (by 30%) and increased mitochondrial depolarization [44] were reported. Deficiencies in perinatal folate intake have been reported in mothers of children with autism [45] in which also abnormalities in their mtDNA copy number have been observed [46].

Considering these two relevant issues not fully addressed in the literature, we studied a Japanese family in which the FGD-affected patient has a novel homozygous substitution (c.644T > C; F215S) at exon 5 in *NNT* [47]. In silico analysis predicted that F215S substitution has the potential to disrupt the NAD binding site, decreasing NNT activity to levels that could reach pathological consequences; however, the impact of this mutation on NNT activity, ROS-mediated damage, and effect on mtDNA replication has not been reported in this or in any other cases of FGD. The aim of this study was to assess (a) the activity of NNT in lymphocytes from this patient and his parents, which are homozygous and heterozygous carriers for this point mutation, respectively, and (b) the putative impact of this mutation on cellular oxidative/nitrative stress and mtDNA replication.

## 2 Material and methods

### 2.1 Subjects

The patient's and parents' genotyping has been published before [47]. The patient (male, 21 y at the time of blood sample collection) was clinically diagnosed with FGD at 19 months of age based on (i) hyperpigmentation, (ii) primary hypocortisonism without increased 17-OH progesterone, (iii) no definitive mineralocorticoid deficiency, and (iv) normal male sex development. Mutation analysis performed at the age of 17 y revealed that this patient carries a homozygous F215S mutation in *NNT* [47]. Proband's mother (52 y) and father (57 y), with no FGD, are both heterozygous for this mutation. This substitution was absent from 120 Japanese control subjects and was not registered in public databases including Japanese SNP Database (http://snp.ims.u-tokyo.ac.jp/). The patient is under oral hydrocortisone replacement therapy. Adrenocorticotropic hormone (ACTH) levels were 5.0, 7.4 and 19.6 pg/ml for patient (under glucocorticoid replacement therapy), father and mother, respectively.

Blood lactic acid [10.8 mg/dl (4–17)], and lactic acid-to-pyruvate ratio were within normal range [10.8 (< 15)]. For this study, peripheral lymphocytes from the patient and his parents were studied. Four of the samples used as controls for NNT activity, NNT protein expression, nitrated tyrosine, and cytochrome *c* oxidase activity were from race- and sex-matched individuals, in which 2 matched the age of the proband (25 and 29 y old) and the other 2 matched the parents' age (54 and 58 y old) and sex. Data from 50 healthy individuals were used for the comparison of citrate synthase activity, mtDNA copy number and mtDNA deletions. Informed consent was obtained in all cases. This study was approved by the Institutional Review Board Committee at Hamamatsu University School of Medicine.

### 2.2 Animals

Adult [55] C57BL/6NJ and C57BL/6J (6–7 month old) mice were obtained from Jackson Laboratories (Sacramento, CA). All procedures were conducted in strict compliance with the policies on animal welfare of the National Institutes of Health and the University of California at Davis (stated in the “Guide for the Care and Use of Laboratory Animals,” Institute of Laboratory Animal Resources, National Research Council, 1996 edition), and approved by the University of California at Davis Animal Care and Use Committee.

### 2.3 Genotyping mice for NNT

All mice utilized in this study were genotyped for the wild-type or mutated *NNT* as described [39]. Genomic DNA was isolated from 5 mg of hindbrain as previously described [56]. Concentration and purity of DNA was measured at an absorbance of 260 nm and 280 nm on a Tecan infinite M200 Nanoquant (Tecan, Austria).

### 2.4 Chemicals and biochemicals

Tris, EDTA, HEPES, MOPS, NaCl, palmitoylCoA, rotenone, sucrose, 3-acetylpyridine adenine dinucleotide (APAD), and β-NADPH were all purchased from Sigma (St. Louis, MO). All reagents were of analytical grade or higher.

### 2.5 Isolation of mitochondria-enriched fraction from human peripheral lymphocytes

Peripheral lymphocytes were isolated from 10-ml of venous blood of each subject collected with heparin under standardized conditions using Lymphoprep density gradient centrifugation (Axis-Shield, Oslo, Norway). Collection of lymphocytes was performed by aspiration, and resuspending them in cryopreservation solution (CELLBANKER^TM^ 1 Nippon Zenyaku Kogyo Co., Ltd, Japan) containing DMSO and newborn calf serum. Cell pellets were kept at − 20 °C until analysis. Mitochondrial membrane-enriched fraction was isolated as reported by others with modifications [48]. Briefly, isolated lymphocytes were washed with PBS and centrifuged at 300 *g* for 15 min, then suspended at a ratio of 10 × 10^6^/ml in a hypotonic medium (10 mM HEPES, pH 7.6); after 4 min on ice, the suspension was centrifuged at 1500 ×*g* for 10 min at 4 °C. The supernatant was centrifuged at 10,000 ×*g* for 20 min to precipitate mitochondrial membranes. These mitochondrial enriched-membrane proteins were resuspended in 200 μl of 0.25 M sucrose, 2 mM EDTA, pH 8.0 and kept at − 20 °C until analysis.

### 2.6 Non-energy linked NNT activity

Spectrophotometric assessment of NNT activity was performed essentially as described by others [49], [50]. The reaction mixture was composed of 50 mM Tris (pH 8.0), 300 μM APAD (3-acetylpyridine adenine dinucleotide; Sigma-Aldrich), an analog of NAD^+^, 300 μM β-NADPH and 10 μM rotenone. APAD was used instead of NAD^+^ because this compound has a different absorption spectrum from NADPH and NADH. The reaction was initiated by the addition of 100–150 μg homogenized mitochondrial enriched-membrane proteins (20 μl) obtained by 3 cycles of freeze–thawing followed by a 5-s sonication with a Fisher 550 sonic dismembrator (intensity 2.5) [51]. Changes in absorbance were monitored at 375 nm for 3 min. Rates of NNT were converted to nmol APAD reduced/min using the extinction coefficient (5.1 mM^− 1^ × cm^− 1^) and normalized to protein, which was determined using the BCA assay. To provide experimental controls for NNT-mediated APAD reduction, homogenized mitochondria were incubated for 5 min with 1 mM palmitoylCoA, competitive inhibitor of NNT [52].

### 2.7 Evaluation of mitochondrial enzymatic activities

Evaluation of mitochondrial enzymes was carried out in either total human lymphocyte homogenates or in mouse mitochondria isolated from cerebellum by differential centrifugation as previously described [57], [58]. Activities of NQR (Complex I), CCO (Complex IV), ATPase and citrate synthase were evaluated as described before using a Tecan Infinite M200 microplate reader [53].

### 2.8 Evaluation of mtDNA copy number and deletions

Genomic DNA was isolated from either human cell pellets (0.6 × 10^6^ cells) or murine cerebellum using the Gentra Puregene Cell and Tissue Kit (Qiagen cat no: 158388) following the manufacturer's recommendations for body fluid samples and tissue samples respectively [46], [56]. Purity of DNA, measured at 260 nm and 280 nm on a Tecan infinite M200 Nanoquant (Tecan, Austria), was > 1.9 for all samples. Following DNA extraction, changes in mtDNA copy number and deletions were evaluated as described in details elsewhere [46], [56]. Relative mtDNA copy number per cell was assessed by a comparative Ct method, using the following equation: mtDNA/nDNA = 2^− ΔCt^, where ΔCt = Ct_mitochondrial _− Ct_nuclear_. Each sample was analyzed in triplicates. The gene copy number of cytochrome ND1 was normalized by a single copy nuclear gene (pyruvate kinase) to reflect the mtDNA copy number per cell whereas the ratios of CYTB or ND4 copy number normalized to that of ND1 reflected mtDNA deletions.

### 2.9 Protein determination

Protein evaluation in samples was performed with the Pierce BCA protein assay (Thermo Scientific, Waltham MA).

### 2.10 Western blotting

Western blotting procedures were performed essentially as described before [53], [54] with the modifications indicated below. Protein extracts from total cell homogenates were concentrated and partly delipidated by acetone precipitation, through the addition of 4 volumes of − 20 °C acetone to each homogenate. Acetone-containing mixtures were vortexed and placed at − 20 °C for 24 h. Samples were then centrifuged at 16,000 ×*g* for 10 min at 4 °C. After pouring off the supernatant, the pellet was resuspended and washed two more times with − 20 °C acetone, spinning each wash at 16,000 ×*g* for 10 min at 4 °C. After removing the supernatant from the final wash, the samples were placed in the SpeedVac for 15 min to remove residual acetone. Samples were resuspended in RIPA buffer (25 mM MOPS, 150 mM NaCl, 1 mM EDTA, 1% NP-40, 0.1% SDS, 1% DOC, pH 7.5) and the protein concentration was evaluated using a BCA Protein assay kit (Pierce #23227). Proteins pellets were denatured in NuPAGE sample buffer (Life Technologies) plus 50 mM DTT at 70 °C for 10 min. Forty μg of protein was added to successive lanes in the SDS-PAGE, transferred via iBlot system (Life Technologies) to a 0.2-μm PVDF membrane for 7 min (program 3). Membranes were washed once for 5 min in Tris buffered saline, followed by blocking in Odyssey Blocking Buffer (LI-COR # 927-40000) for 1 h and then incubated with primary antibodies to COXIV (Cell Signaling, 1:1000), GAPDH (Proteintech, 1:1000), NNT (Proteintech, 1:750), and nitrotyrosine (Millipore, 1:1000), overnight at 4 °C. Membranes were washed 4 × for 5 min with TBST (150 mM NaCl, 25 mM Tris, pH 7.4, 0.1% Tween-20) and then incubated with IRDye 800CW goat anti-rabbit antibody (LI-COR) or 680 goat anti-mouse antibody (LI-COR), diluted 1:10,000 for 1 h at room temperature. After washing the membranes 3 × for 10 min with TBST, proteins were visualized with an Odyssey infrared imaging system (LI-COR). Images were analyzed with the Odyssey V3.0 software and the band intensities expressed as arbitrary fluorescence units (AFU).

### 2.11 Statistical analyses

Data from patient and parents were obtained from at least duplicates run on the same day. Other details were indicated under Table 1, Table 2.

### 2.12 Molecular modeling

The 3D structure of the NNT was obtained through modeling of the mature, primary sequences of the human WT and F215S NNT [59], [60] as indicated before [61]. The 3D structure was further refined by using ChemDraw. All pdb files were visualized using PyMol 1.4.1 [62].

## 3 Results

### 3.1 NNT activity in peripheral lymphocytes from carriers follows a gene–dose response

The non-energy linked activity of NNT was evaluated in mitochondria-enriched fractions from lymphocytes obtained from a 21 y old patient (carrying a homozygous F215S NNT mutation), his parents (heterozygous for this mutation) and controls. The NNT activity values from control (regardless of age or sex) in peripheral lymphocytes, expressed per mg of mitochondrial protein, were 27 to 62 nmol × (min × mg)^− 1^, consistent with those reported before for mitochondria from tissues with relatively low endogenous NNT activity [e.g., prostate, seminal vesicles, spleen, and testis [63]] and within values of other solubilized mitochondrial transhydrogenases [30–80 nmol × (min × mg protein)^− 1^ [64], [65], [66]]. No difference in NNT activity was observed between younger and older controls (respectively 1.31 ± 0.05 and 2.05 ± 0.5; *p* = 0.177). The NNT specific activity [defined as the activity expressed as nmol product × (min × 10^6^ cells)^− 1^ normalized by the NNT protein levels] in the patient's sample was 31% of the mean control value and 56% of his parents (Table 1), both values outside the 95%CI. The average NNT activity of the parents was not different from each other but, in both cases, lower than controls average (61% of controls). Thus, these results indicated that the decrease in NNT activity followed a gene–dose dependency.

**Fig. 1:**
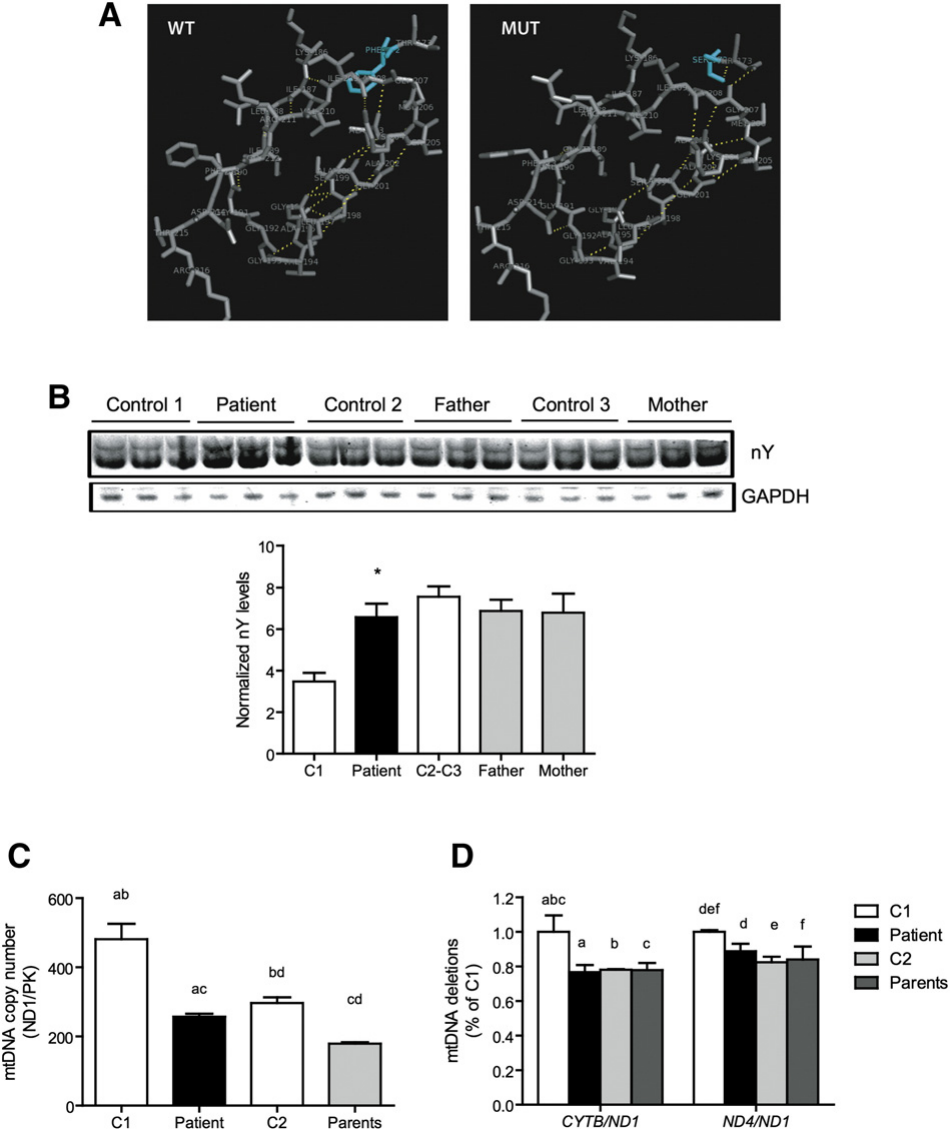
A. In silico modeling of NNT structure. The 3D images were obtained through modeling of the mature primary sequences of the human wild-type (WT) and F215S mutant NNT (MUT), as described in the Material and methods section (subsection 2.12). Protein backbone is in gray, amino acid of interest shown in blue and H-bonds are in yellow. 1B. Nitration of tyrosine residues in lymphocytes. Representative Western blot image of proteins with nitrated tyrosine residues in lymphocytes from patient, his parents and age-matched controls (C1 = patient's age-matched controls, 25 years old; C2 and C3 = parents' age-matched controls, 54 and 58 years old respectively). Western blots were carried out as described in the subsection 2.5 of Material and methods. The lanes were loaded with equal cell concentrations and the densitometry of the nitrotyrosine bands was normalized by GAPDH. The results of the quantification are shown in the lower panel. *p < 0.05 vs. C1. 1C. mtDNA copy number in lymphocytes from homozygous and heterozygous carriers and age-matched controls. The mtDNA copy number per cell and deletions were estimated by qPCR as described under Material and methods (subsection 2.9) and expressed as fold-change relative to the youngest control (C1). ANOVA followed by Bonferroni post-hoc test: a, b = *p* < 0.01; c, d = *p* < 0.05. 1D. mtDNA deletions in lymphocytes from homozygous and heterozygous carriers and age-matched controls. Deletions were evaluated at the segments encoding for *CYTB* (cytochrome *b*), and *ND4* (NADH dehydrogenase subunit IV). Due to the limited amount of samples from the parents, these samples were combined for the extraction of gDNA. As a result, the evaluation of mtDNA copy number and mtDNA deletions were performed on these pooled samples. ANOVA followed by Bonferroni post-hoc test: a, e, f = *p* < 0.01; b, c, d = *p* < 0.05.

To visualize the F215S mutation in the NNT protein, the mature and mutated proteins were modeled in silico (Fig. 1A). From the 4 full primary sequences of NNT (human, mouse, bovine and rat) available in the protein database, F215 is present in a highly conserved segment not part of the predicted NAD binding site (residues 229–259). However, the phenyl ring is facing a hydrophobic pocket lined with Ala, Ile, and Leu residues, which should bind part of the NAD moiety. When F215 is replaced by S, new H-bonds are formed between S215 and the adjacent T216, disrupting the H-bonds between residues 250–252. As a result, changes in size and shape of this pocket occur, possibly affecting the H^+^ transfer [26], and in turn its transhydrogenase activity. Thus, the in silico analysis is consistent with the significant NNT activity loss induced by the F215S mutation.

### 3.2 Increased nitrative stress in lymphocytes from FGD patient but not in heterozygous carriers

Given the putative role of NNT at providing NADPH for several processes including mitochondrial antioxidant defenses [35], [37], the oxidation/nitration of lymphocytic proteins was evaluated by Western blotting using antibodies against nitrotyrosine. Tyrosine nitration was mostly evident in the patient's lymphocytes at bands with MW of 55 and 58 kD (Fig. 1B). At these bands, protein nitration in the patient's lymphocytes, expressed as arbitrary fluorescence units (AFU) and normalized by GAPDH, was 10.1 ± 2.9 (mean ± SEM), 2.2-fold higher than aged-matched control (*p* = 0.04). In the father's and mother's lymphocytes, tyrosine nitration levels were 6.2 ± 0.4 and 7.7 ± 3.0, respectively, within the range obtained with age-matched controls and not different from the nitration observed in the proband's lymphocytes.

### 3.3 Lower mitochondrial mass in mutation carriers but lower OXPHOS only in FGD-affected patient

In parallel experiments, to evaluate the effect of the *NNT* mutation on the mitochondria number and oxidative phosphorylation (OXPHOS) capacity, we assessed the activities of citrate synthase, as a relatively stable marker of mitochondrial mass [67], and cytochrome *c* oxidase or Complex IV (CCO), the terminal oxidase of the electron transport chain. The activity of citrate synthase was lower in both patient and parents compared to that in controls, regardless of age (20 to 30% of control values; Table 1) suggesting a lower mitochondria number/cell. The activity of Complex IV, normalized by citrate synthase, was significantly lower in the patient's lymphocytes with no differences between that in the parents and controls (Table 1). The protein expression values of the subunit IV of CCO normalized to GAPDH for the patient and his parents were within the 95% CI calculated with control values and, as such, not different from controls (for COXIV/GAPDH mean ± SD = 5.1 ± 0.1 for the patient, 3.6 ± 0.1 for his parents; range for controls = [1.3, 11.9]). Thus, these results suggested that the lower CCO activity could not be attributed to a defective synthesis and/or import of the nuclearly-encoded subunit IV and, possibly that of other nuclear DNA (nDNA)-encoded mitochondrial proteins, but to a lower contribution of the mtDNA-encoded CCO subunits, which include the CCO catalytic subunits.

Taken together, these results suggested that the presence of at least one *NNT* mutated allele leads to a significant lower mitochondrial mass, a situation that is enhanced specifically in the patient by the lower CCO specific activity.

### 3.4 mtDNA copy number and deletions in FGD

Given the NADPH dependency of DHFR (and DHFRL1; [68]), enzyme of the dTMP pathway required to maintain mtDNA integrity [40], the mtDNA copy number per cell and mtDNA deletions were evaluated in the patient's and his parents' compared to age-matched control lymphocytes (Fig. 1C and D).

The mtDNA copy number in the patient's lymphocytes was 56% lower than the mean values for age-matched controls (Fig. 1C), accompanied by mtDNA deletions at various segments of the mtDNA (CYTB/ND1: patient = 0.77 ± 0.04, age-matched controls = 1.00 ± 0.09, *p* < 0.01; ND4/ND1: patient = 0.89 ± 0.04, age-matched controls = 1.00 ± 0.01, *p* < 0.05). The extent of these deletions was comparable to that observed in individuals 30 years older than the patient, including his parents (Fig. 1D). The parents' mtDNA copy number was 40% lower than the mean age-matched control value (Fig. 1C); however, the mtDNA deletions in the parents' lymphocytes were within those expected for an older group (Fig. 1D). Of note, a statistically significant difference in mtDNA copy number was observed even between younger and older controls, consistent with some findings reporting decreased mtDNA copy number as an aging-related outcome [69], [70].

The lower mtDNA copy number (as observed in patient and his parents), lower mitochondrial mass (patient and his parents), and significant increased mtDNA deletions (patient) compared to the increased protein nitration (patient) seem to point to a phenotype different to that solely defined by increased oxidative stress. Indeed, the mitochondrial outcomes of the patient were aligned to those reported for folate deficiency in which deficits in OXPHOS and increased mtDNA deletions have been observed [43], [44], [71]. Moreover, as reported for folate deficiency [72], a lower peripheral lymphocytic yield was obtained in all carriers compared to controls (range of controls 10 to 20 × 10^6^ cells/8–12 ml blood; for carriers in 10^6^ cells: patient 8.2, father 4.5 and mother 6.4). The lower lymphocyte yield observed in parents, relative to the patient, could be explained by an age-dependent effect added to the NNT heterozygosity.

### 3.5 Comparison of mitochondrial outcomes in FGD to a murine model of NNT deficiency

To ascertain similarities between the patient and a murine model of NNT deficiency, mitochondrial outcomes were evaluated in cerebella from adult C57BL/6J [*NNT*^−/−^ carrying a spontaneous *NNT* mutation resulting in negligible NNT activity [37]] and C57BL/6NJ (*NNT*^+/+^) substrains. Although other genetic differences have the potential to contribute to the phenotypic differences between these substrains [39], [73], the loss of NNT activity in cerebellum from *NNT*^−/−^ mice was accompanied by a lower mitochondrial mass (as judged by CS activity; ~ 70% of controls) as well as lower Complex I, IV and V activities normalized to citrate synthase (in average by 34% of controls; Table 2). The mtDNA copy number was significantly lower in *NNT*^−/−^ (49% of control values; Table 2) accompanied by significant increases deletions at the segments encoding for *CYTB* (30%) and *ND4* (9%). Thus, taken these results together, the mitochondrial outcomes observed in *NNT*^−/−^ mice were similar to those from the patient's lymphocytes, and both characterized by lower mitochondrial mass, lower OXPHOS capacity and increased mtDNA deletions.

## 4 Discussion

In this study, for the first time we characterized mitochondrial outcomes in lymphocytes from a patient affected with FGD carrying a novel, homozygous *NNT* F215S mutation. A mechanism linking defects in NNT and glucocorticoids deficiency has not yet been established; however, considering that NADPH-dependent mitochondrial processes such as (i) ferredoxin reductase and ferredoxins for biosynthesis of iron-sulfur clusters [13] and steroid hormones [22], [23], [24], and (ii) adrenodoxin oxidoreductase, serving as the first electron transfer protein in all the mitochondrial P450 systems (including cholesterol side chain cleavage in all steroidogenic tissues, steroid 11-beta hydroxylation in the adrenal cortex, 25-OH-vitamin D3-24 hydroxylation in the kidney, and sterol C-27 hydroxylation in the liver [74], [75]), it is conceivable that the reducing equivalents required for these processes are supplied by NNT.

Lymphocytes from the FGD patient analyzed in this study exhibited a significant (by 70%) loss-of-function activity of NNT, consistent with the lower H^+^ transfer predicted by the 3D protein model. The lower NNT activity was accompanied by deficits in OXPHOS capacity, lower mitochondrial mass, increased mtDNA deletions and increases in protein nitration. When the patient's outcomes were compared to his parents', clinically healthy but heterozygous carriers for this mutation, the results suggested that (i) at ≤ 60% of NNT activity, mitochondrial biogenesis (citrate synthase activity) and/or mtDNA replication (mtDNA copy number) are affected because these parameters were abnormal in individuals carrying one or both mutated alleles; and (ii) ≤ 30% NNT activity, deficits in other outcomes (mtDNA deletions, protein nitration, OXPHOS capacity) are triggered in line with (a) the minor diagnostic criteria for mitochondrial respiratory chain disorders [mitochondrial activities between 20 and 30%; [76]], (b) the results obtained with murine cerebellar mitochondria from C57BL/6J (*NNT*^−/−^) vs. C57BL/6JN (*NNT*^+/+^) substrains, and (c) those published with negligible NNT activities and increased oxidative stress [34].

Our results support the concept that proton-pumping transhydrogenases constitute one of the main sources of mitochondrial NADPH in eukaryotes (35 to 45% of the total NADPH is provided by both NNT and the pentose phosphate pathway [30], [77]), followed by the NADP^+^-isocitrate dehydrogenase (20 to 25%) and the mitochondrial NAD(P)^+^-malic enzyme [~ 10% [30], [77], [78], [79]]. However, this role is not only relevant to adrenocortical cells [15], but also to other cells (not necessarily associated with steroidogenesis) where *NNT* is expressed to varying degrees [80]. While most of the work in the field has highlighted the involvement of NNT, directly or indirectly, in the maintenance of antioxidant defenses [20], [30], [34], [37], [81], [82], the apparent discrepancy between the NNT thresholds for mtDNA replication and/or mitochondrial biogenesis and that for oxidative stress-mediated outcomes (protein nitration, mtDNA deletions) suggests that NNT is not the rate limiting step in the ROS detoxification system [as glucose-6-phosphate dehydrogenase is in PC12 cells [83]], in which other NADPH sources overlap and complement mitochondrial NADPH production [30], [77], [78], [79]. In support of this concept, it has been reported that tissues from *NNT^−/−^* mice with no detectable NNT activity showed only a 33% decrease in the [GSH]/[GSSG] ratio [37], indicating that the majority (~ 70%) is accounted for by other NADPH sources. Moreover, the main involvement of NNT as part of the antioxidant response has been challenged by the higher susceptibility of *NNT*^+/+^ mice vs. *NNT^−/−^* to acetaminophen- and concanavalin A-induced liver injury [84] and to cerulein-induced chronic pancreatitis [85], biological models of organ injury in which mitochondrial oxidative stress seems to play a pathological role [86], [87], [88], [89].

**Fig. 2:**
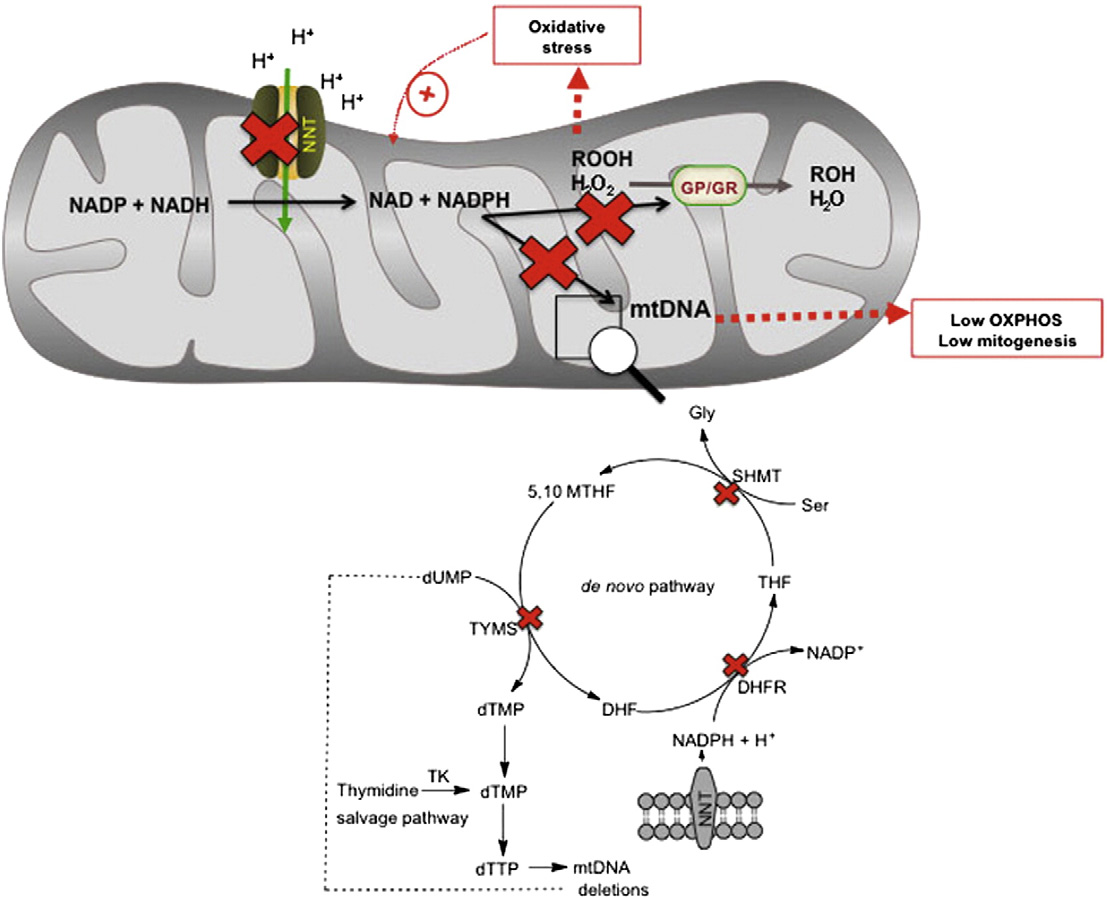
Molecular pathways linking mutant NNT to oxidative stress, deficits in mitochondrial energy production and decreases in mtDNA integrity. NNT constitutes one of the main sources of mitochondrial NADPH in eukaryotes. The NNT loss-of-function results in lower NADPH production, resulting in deficits in antioxidant defenses (via glutathione peroxidase/reductase), decreased mtDNA integrity (copy number and deletions), and lower mitochondrial mass. We hypothesized that NNT may act as the main provider of NADPH for de novo thymidylate synthesis. Deficits in dTTP could affect the maintenance of mtDNA integrity in the de novo thymidylate biosynthesis, impairing the synthesis of dTMP required for mtDNA replication and increase mtDNA deletions. Abbreviations: SHMT, DHFR, TYMS and TK are serine hydroxylmethyl transferase, dihydrofolate reductase, thymidilate synthase and thymidine kinase, respectively. DHF, THF, 5,10-MTHF are dihydrofolate, tetrahydrofolate and 5,10-methyl-tetrahydrofolate.

Then, the distinctions between homozygous and heterozygous carriers seem to originate from differences in either the NADPH threshold for these processes or the rate of these reactions in vivo, i.e., the antioxidant response vs. mtDNA replication/repair pathway. The first alternative seems unlikely based on the relatively low *K_m_* of NADPH for both glutathione reductase (5 to 25 μM [90], [91], [92]) and DHFR (or DHFRL1) (3–4 μM [68]), even with a 30% residual NNT activity such as that observed in the FGD patient reported in this study. Alternatively, NNT may act as the main provider of NADPH for thymidylate synthesis (Fig. 2). Deficits in dTTP could affect the maintenance of mtDNA integrity in the de novo thymidylate biosynthesis, impairing the synthesis of dTMP required for mtDNA replication [40], [43], [44], [71], [93] and increase mtDNA deletions, as observed in mice lacking *UNG* undergoing folate deprivation [71]. Indeed, the lower mtDNA copy number observed in FGD could be explained by a prolonged replication pause [94] resulting from altered nucleotide precursor pool as mtDNA instability increases in the presence of a limiting dTTP pool as it has been reported for folate deficiency [43], [44], [71], [93]. In line with this reasoning, the deficiencies reported in perinatal folate intake in mothers at risk of having a child with autism [45] could explain the differences in copy number and deletions observed in peripheral blood cells in children with autism [46], [95].

Considering that the mtDNA deletions observed in this patient with a homozygous *NNT* mutation were similar to those expected for individuals 30 years older, it highlights the role that mtDNA cumulative damage could have at enhancing and/or extending the morbidity of FGD to symptoms observed in mitochondrial disorders (such as ketosis, muscle weakness, progressive neurodegeneration), favored by deficient antioxidant responses aided by the replicative advantage of large-scale deletions [96], [97], accumulation of mtDNA damage over time [69], [70] and by the decline in *NNT* gene expression with age [98]. Thus, from the clinical point of view, it is important to emphasize the need for a long-term systematic medical follow-up of FGD patients.

Finally, it is important to emphasize the parallel between this family with a point mutation in *NNT* and that of a family with a homozygous mutation in *MCM4/PRKDC* presenting FGD features and atypical Fanconi type DNA breakage disorder [99]. These genes, shown to function in mitochondria [100], [101], are involved in the ataxia-telangiectasia-mutated/ATM-Rad 3-related DNA repair pathway. Then pathogenic mutations in *NNT* (this study), *MCM4*/*PRKDC* [99] and *TXNRD2* [19] highlight the morbidity of a dysfunctional mitochondrial antioxidant/repair pathway in steroidogenic as well as in non-steroidogenic tissues. Deficits in the mitochondrial antioxidant/repair pathway (along with disease modifiers, such as genetic background and environmental triggers) can contribute to the morbidity of the FGD syndrome as evidenced by those clinical outcomes also shared by other mitochondrial disorders [e.g., failure to thrive, recurrent infections; [15], [76], [103], [104], [105]]. It also indicates that mtDNA damage might be a common pathological condition in a subgroup of FGD patients with defective responses to DNA damage [102].

## 5 Conclusions

This study provides for the first time an insight into the role of NNT in the maintenance of mtDNA and underlines the relevance of assessing NNT activity to predict its impact on mitochondrial outcomes to design a rationale therapy that minimizes mitochondrial deficits while receiving supplementation with corticoids.

## Figures and Tables

**Table 1:** Mitochondrial outcomes in lymphocytes from patient and controls.

Outcome	Patient	Mother	Father
NNT activity/NNT protein levels (× 1000)	**0**.**57 ± 0**.**02**	**1**.**00 ± 0**.**003**	**1**.**04 ± 0**.**03**
Range	[1.27, 2.40]		
Nitrotyrosine (normalized by GAPDH)	**10**.**1 ± 2**.**9**	7.7 ± 3.0	6.2 ± 0.4
Range	[3.4, 5.6]	[4.3, 10.6]	
Citrate synthase activity	**2**.**1 ± 0**.**1**	**1**.**7 ± 0**.**1**	**4**.**6 ± 0**.**8**
95%CI	[6,12]		
CCO/CS activity	**1**.**8 ± 0**.**4**	4.2 ± 0.5	2.3 ± 0.3
Range	[2.1, 4.4]		

Bold numbers indicate values outside either the 95%CI (calculated with control values) or Range (when the number of control individuals for a particular outcome was < 4, the range represents the lowest and highest values obtained for that outcome). When no statistically significant difference was observed between younger and older control individuals only one combined 95%CI or range was reported instead of two. The 95%CI for citrate synthase activity was calculated using data from healthy individuals aged 20 to 30 y (*n* = 38) and 40 to 60 y (*n* = 12) that matched the age of the parents. NNT activity, CCO activity and CS activity were all expressed as nmol × (min × 10^6^ cells)^− 1^. NNT activity was then normalized to the intensity of the NNT band obtained by Western blots to express the specific activity. AUD, arbitrary units of density of the nitrated protein band normalized to an unknown protein used as loading control.

**Table 2:** Mitochondrial outcomes in cerebellar mitochondria from *NNT*^−/−^ and *NNT*^+/+^ mice.

Outcome	*NNT*^+/+^	*NNT*^−/−^	*p*-Value^a^
Citrate synthase activity	280 ± 10	200 ± 1	< 0.05
NQR/CS	0.45 ± 0.08	0.21 ± 0.02	0.05
CCO/CS	0.34 ± 0.02	0.29 ± 0.02	0.05
ATPase/CS	3.3 ± 0.6	2.14 ± 0.07	0.05
mtDNA copy number/cell	273 ± 10	133 ± 4	< 0.001
mtDNA deletions *CYTB*/*ND1* (%)	< 1	30 ± 4	0.004
*ND4/ND1* (%)	< 0.5	9 ± 2	0.004

Citrate synthase, NQR, CCO and ATPase activity were all expressed as nmol × (min × mg protein)^− 1^, and the last 3, normalized to citrate synthase. Data are reported as mean ± SEM of experiments ran in triplicates with tissues obtained from 4 *NNT*^+/+^ and 6 *NNT*^−/−^ mice. Mitochondrial DNA copy number and deletions were calculated as described in the Material and methods section. The average *CYTB/ND1* and *ND4/ND1* control ratios were taken as 100% (no deletions) and *NNT*^−/−^ mitochondrial gene ratios were expressed as percentages of controls. The differences between 100% and the *NNT^+/+^* or *NNT*^−/−^ percentages were taken as the % of deletions for that particular mtDNA segment.

a The *p*-values were calculated by using the 2-tailed Student's *t* test and considered significant if *p* < 0.05.
